# Selective vulnerability in α-synucleinopathies

**DOI:** 10.1007/s00401-019-02010-2

**Published:** 2019-04-20

**Authors:** Javier Alegre-Abarrategui, Katherine R. Brimblecombe, Rosalind F. Roberts, Elisavet Velentza-Almpani, Bension S. Tilley, Nora Bengoa-Vergniory, Christos Proukakis

**Affiliations:** 1 Centre for Neuroinflammation and Neurodegeneration, Division of Brain Sciences, Faculty of Medicine, Imperial College London, Du Cane Road, London, W12 0NN UK; 2 Department of Physiology, Anatomy and Genetics, Oxford Parkinson’s Disease Centre, University of Oxford, South Parks Road, Oxford, OX1 3QT UK; 3 Montreal Neurological Institute, McGill University, 3801 Rue University, Montreal, QC H32 2B4 Canada; 4 Department of Movement and Clinical Neurosciences, UCL Queen Square Institute of Neurology, University College London, Rowland Hill Street, London, NW3 2PF UK

## Abstract

Parkinson’s disease, dementia with Lewy bodies, and multiple system atrophy are neurodegenerative disorders resulting in progressive motor/cognitive deficits among other symptoms. They are characterised by stereotypical brain cell loss accompanied by the formation of proteinaceous aggregations of the protein α-synuclein (α-syn), being, therefore, termed α-synucleinopathies. Although the presence of α-syn inclusions is a common hallmark of these disorders, the exact nature of the deposited protein is specific to each disease. Different neuroanatomical regions and cellular populations manifest a differential vulnerability to the appearance of protein deposits, cell dysfunction, and cell death, leading to phenotypic diversity. The present review describes the multiple factors that contribute to the selective vulnerability in α-synucleinopathies. We explore the intrinsic cellular properties in the affected regions, including the physiological and pathophysiological roles of endogenous α-syn, the metabolic and genetic build-up of the cells and their connectivity. These factors converge with the variability of the α-syn conformational strains and their spreading capacity to dictate the phenotypic diversity and regional vulnerability of each disease. Finally, we describe the exogenous and environmental factors that potentially contribute by igniting and modulating the differential pathology in α-synucleinopathies. In conclusion, we think that it is the confluence of this disruption of the cellular metabolic state and α-syn structural equilibrium through the anatomical connectivity which appears to initiate cascades of pathological processes triggered by genetic, environmental, or stochastic events that result in the “death by a thousand cuts” profile of α-synucleinopathies.

**Electronic supplementary material:**

The online version of this article (10.1007/s00401-019-02010-2) contains supplementary material, which is available to authorized users.

Parkinson’s disease (PD), dementia with Lewy bodies (DLB), and multiple system atrophy (MSA) belong to the group of devastating neurodegenerative disorders known as α-synucleinopathies. They share multiple characteristics such as a preference for affecting the motor and/or cognitive spheres and an orderly recruitment of brain regions to the disease in a stereotyped manner, pathologically featuring brain cell loss accompanied by proteinaceous aggregates of the protein α-synuclein (α-syn). Different neuroanatomical regions, and within these regions different neuronal and glial populations, show a differential vulnerability to dysfunction and cell death in α-synucleinopathies, resulting in phenotypic diversity. The way in which disease starts and progresses and the relative dysfunction of neuronal circuitries is probably the resulting combination and interplay of multiple factors including (1) intrinsic cellular properties in the affected regions, such as the normal and aberrant properties of the endogenous α-syn protein, the metabolic and genetic build-up of the cells, and their connectivity, (2) the existence of different α-syn conformational strains and a differential facilitation, permissiveness or blocking of each cell type in generating and/or transferring them to other cells, thereby generating specific neuroanatomical routes for their spread, and (3) putative exogenous and environmental factors acting as triggers or modulators of pathology.

## Intrinsic cellular properties underlying selective vulnerability

The differential vulnerability of cellular populations and regions to degeneration in α-synucleinopathies is in no doubt influenced by intrinsic cellular properties in the affected regions, such as the normal and aberrant properties of the endogenous α-syn protein, their metabolic and genetic build-up and their connectivity.

### Function and dysfunction of endogenous α-syn

Here, we discuss the molecular nature of α-syn which give rise to its physiological and pathophysiological properties and the cellular characteristics of nuclei affected in α-synucleinopathies which may predispose them to degeneration.

#### The structural basis of α-syn

The physiological and pathological nature of α-syn is determined by its molecular and structural properties. The complexity of α-syn pathology mirrors its ability to form a broad range of structures, its diverse post-translational modification status, and its ability to associate with both lipid and protein partners. Understanding how α-syn affects specific biological pathways and downstream physiological functions provides insights into the differential cellular and regional vulnerabilities in α-synucleinopathies [108, 150, 158].

α-Syn is a small protein (140 amino acids) expressed throughout the brain and at lower levels in other tissues including gut, heart and blood cells. α-Syn consists of 3 domains: (1) the amphipathic N-terminal domain contains 7 conserved but imperfect repeats of 11 amino acids, each containing the consensus sequence KTKEGV [59]. The N-terminus domain is thought to be important for α-syn-lipid interactions, whereby the repeats promote α-helix over β-sheet structure of the protein, particularly when exposed to negatively charged lipids [11]. (2) Residues 61–95 form the core region, known as non-amyloid-β component (NAC). NAC is important for fibril formation and aggregation of α-syn due to its propensity to form cross-beta sheets. (3) The C-terminus tail is highly acidic, and proline rich resulting in a disordered, random coil structure. Interactions between C-terminus, and NAC domains prevent α-syn aggregation, consistent with the high structural homology of C-terminus with heat-shock proteins capable of limiting α-syn aggregation [30].

While mostly thought to adopt an unfolded monomeric structure, α-syn can exist in multiple molecular weight species including monomeric, oligomeric, and fibrillar structures. These species are believed to assemble in a stochastic manner and to potentially inter-convert and exist in an equilibrium in which the concentration and lifespan of each conformation would depend on multiple physical and chemical factors in the cellular milieu [134]. Factors which shift the structural equilibria ultimately result in the assembly of different physiological and pathological strains.

#### The physiological role of α-syn

α-Syn in the brain is predominantly pre-synaptically located, where it has a propensity to bind to curved membranes including vesicles and is often associated with known components of the exocytotic machinery [33, 59]. α-Syn accumulation in pre-synaptic terminals causes a synaptopathy thought to ultimately lead to a dying back-like mechanism of neurodegeneration [27]. Additional pathogenic mechanisms of α-syn seem to derive from its intimal physiological association with mitochondria, endoplasmic reticulum (ER), and lysosomal structures [32, 174], where it interacts with a range of protein and lipid partners.

The effect of α-syn on the release of several neurotransmitters including dopamine (DA), noradrenaline, serotonin, and glutamate has been explored [87, 98, 167]. However, even for a given neurotransmitter, α-syn has been reported to have wide-ranging effects. For example, α-syn has been reported to both decrease, increase and have no effect on DA release depending upon the species of α-syn and the region of recording [87, 98]. These varying effects illustrate the complex interaction between α-syn and individual neurotransmitter systems. The mechanisms underlying α-syn effects on neurotransmission have undergone much investigation in part driven for the insights into possible pharmacological interventions. α-Syn has been shown to increase the voltage-gated calcium channel (VGCC) function, e.g., by decreasing raft partitioning of Cav2.2 channels [152], which could contribute to the facilitatory effects of α-syn on neurotransmitter release. In contrast, α-syn assemblies have been found to bind N^+^/K^+^ATPase (NKA), leading to areas of low NKA density, affecting membrane repolarisation, which may decrease release [158]. The Soluble *N*-ethylmaleimide-sensitive Factor (NSF) Attachment Protein Receptor (SNARE) machinery is a large protein complex which play a key role in membrane fusions, such as the docking of synaptic vesicles with the pre-synaptic membrane in neurons. α-Syn is known to bind to curved membranes and is strongly associated with vesicles, where it is thought to associate precisely with the SNARE machinery for the effective tethering of vesicles [33] with further effects on neurotransmission. The association of α-syn and vesicles, interestingly, appears to result in the release of α-syn into the extracellular space, in a process highly dependent on intrinsic neuronal activities. While the physiological role of this phenomenon is not fully understood, the released α-syn is subsequently taken up by surrounding neurons and glia [99, 136, 188], which is thought to have pathological implications. For example, it is possible that these putative physiological routes are hijacked by pathological species for their own spread.

In addition to interacting with pre-synaptic mechanisms controlling neurotransmitter release, α-syn has also been reported to change cell firing properties. In dopaminergic (DAergic) neurons, mutant and wild-type α-syn have been reported to increase and decrease cell firing, respectively [87, 163]. The increase in DAergic neuron firing by mutant α-syn is due to inhibition of A-type K^+^ channels, whereas the mechanisms of age-related decrease in DAergic neuron firing by wild-type α-syn have not yet been established. However, interactions with NKA, VGCCs, or membrane dynamics could all contribute. It should also be noted that alterations in neuronal firing could also be due to network adaptations, following changes to neurotransmitter release. This chain of events illustrates the ability for α-syn to instigate a cascade of events and the complexity of establishing hierarchy of α-syn pathology when neuronal and α-syn biology is so intricately connected. Nevertheless, it seems plausible that the complex and variable effects of α-syn on neurotransmission may account in part for the phenotypic diversity resulting from the differentially impacted circuitries upon α-syn dysfunction.

Besides the direct implication of α-syn in the synaptic machinery, α-syn is involved in mitochondrial function. Given that generation of energy in the form of ATP is essential for all the stages of neurotransmission, these interactions between α-syn and mitochondria further impact neurophysiology. α-Syn (monomer) increases ATP synthase efficiency [107] and α-syn also affects mitochondrial trafficking and morphology [174]. Rare genetic forms of early onset PD are due to mutations in mitochondrial genes and genes relevant for mitochondrial metabolism (e.g., *PRKN* and *PINK1*, both involved in mitophagy), illustrating an interesting cross-over between mitochondrial function and α-synucleinopathies. Thus, α-syn has wide-ranging effects on mitochondrial function and dysfunction, which has profound implications for neuronal physiology.

#### A putative physiological role for α-syn oligomers/multimeric species

Most of the studies investigating the physiological role of α-syn have focused their attention on the monomeric form. However, recent studies suggest putative physiological roles for oligomers/multimeric species, with a dynamic equilibrium existing between free monomeric species and membrane-bound multimers influenced by the cellular environment. For example, α-syn multimers engage with synaptic vesicles clustering them, thus halting their recycling under physiological conditions [182]. It also seems that the referred physiological function of α-syn in binding to and promoting SNARE complex formation may not be mediated by monomeric α-syn but rather by higher order multimers of α-syn assembling upon membrane binding [33]. The specific nature of these oligomeric/multimeric species engaged in physiological roles, whether they can be precursors within the abnormal aggregation pathway (or rather be off-pathway), and the conditions for any pathological conversion are not fully understood. Recent work has suggested a folded helical tetramer as the physiological α-syn species [52], but other groups have questioned the existence of this tetrameric form [34].

Bona fide prion diseases are characterised by infectious agents composed solely of protein that are transmissible between individuals. The molecular basis of prions is their ability to acquire a self-templating amyloidogenic state. Several human diseases of the brain and other organs are now being shown to be characterised by abnormal protein aggregation that can spread between neighbouring cells and in certain conditions propagate pathology when protein extracts are experimentally inoculated into another individual. Several of the proteins involved in neurodegenerative disorders (e.g., tau, α-syn and β-amyloid) have been shown to have self-templating properties. At least for now, these proteins have not been shown to have all the properties of prions (such as their microbiological transmissibility), and therefore, different names have been proposed to describe them, including prionoids or prion-like proteins.

There are, however, other proteins characterised by self-templating with physiological rather than deleterious consequences. The biochemical process involved seems particularly well suited to produce a long-lasting physiological change upon a transient stimulus [103]. Several are the examples in fungi [45, 57] and in the marine snail *Aplysia*, where the protein cytoplasmic polyadenylation element-binding protein (CPEB) functions to maintain long-term changes in synaptic efficacy through a mechanism involving self-perpetuating aggregation [159]. This mechanism effectively works as a conformational switch for synapse-specific local protein synthesis [160]. Additional examples for physiological functions of self-templating proteins include epigenetic regulation and scaffolding for subcellular organization [110]. Importantly, there are at least two features that theoretically would differentiate these non-pathogenic prion-like processes from the pathogenic ones: (1) that the switch to the self-templating conformation of the protein is initiated by a physiological signal [160] and (2) that the process should be subject to regulatory control of its spatial and temporal propagation [15].

Therefore, we hypothesise that it would not be unthinkable to imagine a physiological role not only for “innocent” α-syn oligomer/multimers, but also for α-syn prionoids (defined as amyloidogenic, self-templating aggregates), perhaps in a process involving a regulated inter-conversion between the different aggregated or monomeric states upon physiological signals, with their dysregulation precipitating disease. These processes could have roles in generating long-term changes at the synapse, as shown with other self-templating proteins. In addition, seeding and propagation of a particularly folded conformation to other parts of the cell and to neighbouring cells could potentially be a resourceful mechanism being exploited physiologically by cells for yet undiscovered but possible functions, from paracrine signalling to a mode of innate defensive mechanism upon cellular stresses such as those derived from infection, traumatic brain injury, inflammation, or ischemia.

#### The toxic properties of α-syn

**Fig. 1:**
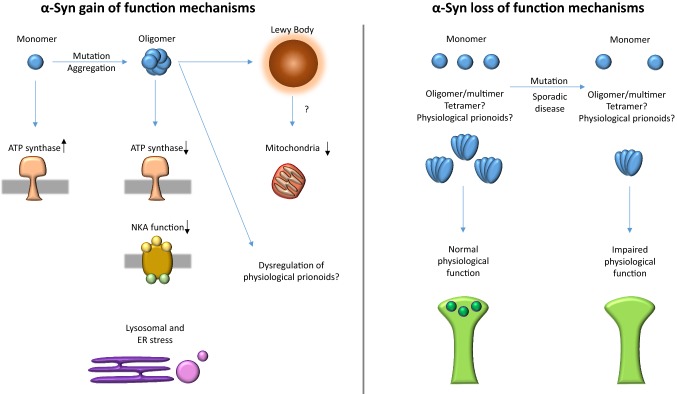
α-Syn potential gain/loss of function mechanisms. While α-syn is able to increase ATP synthase activity in its monomeric state, it reduces its activity in its oligomeric state. Oligomers have also been reported to reduce NKA function and cause lysosomal and ER stress. Mutations in α-syn have also been shown to alter the ratio of monomers with supramolecular species and to impair synaptic function. Similar mechanisms could play a role in sporadic disease. We hypothesise the existence of regulated physiological α-syn prionoids and their dysregulation contributing to disease perhaps by both gain and loss of function mechanisms

Several sources of evidence support that at least part of the toxicity associated with α-synucleinopathies is due to α-syn gaining aberrant properties. However, growing evidence suggests that loss of the required physiological function of α-syn could contribute to neuronal dysfunction and possibly toxicity (Fig. 1). The interplay and proportional contribution of gain and loss of function mechanisms in α-synucleinopathies [14] and what role, if any, they play in generating a differential cellular and regional susceptibility are still not fully characterised.

One of the key discoveries of our understanding of the molecular and pathological mechanisms of PD was the discovery that the A53T mutation, and later, the A30P, E46K, G51D, A53E, and H50Q missense mutations in the α-syn gene (*SNCA*) caused autosomal dominant forms of the disease [126]. It must be said that the pathogenicity of the H50Q has been challenged [18], as it was also found as a rare variant in apparently healthy individuals. Subsequently, *SNCA* copy number variations (CNVs) including duplications and triplications were identified [126]. The autosomal dominant mode of inheritance and the high pathogenic penetrance of most of these mutations seemed to point towards a gain of function mechanism in these familial cases, while the high clinical and pathologic similarities with sporadic disease suggested that such mechanisms could also play a prominent role in the common disease.

What are the ultimate mechanisms mediating this gain of function in *SNCA*-mutated cases? and could they explain part of the differential cellular and regional vulnerability? In the case of CNVs (duplications and triplications), there is a clear dosage effect on the phenotype, with a total of four copies (heterozygous triplication cases, or rare homozygous duplication cases) having a more severe earlier disease than the three copies present in a heterozygous duplication carrier [93]. In the case of the reported missense mutations in *SNCA*, a variety of consequences have been derived from their altered structure. A recurrent finding is that mutations alter the way in which α-syn interacts with itself, changing the relative proportion of the possible species (e.g., monomer, tetramer, oligomer and fibril), with most of these missense mutations promoting fibrillar [63] and/or oligomeric forms of the protein. In the case of sporadic disease, it is not established what is the initiating (e.g., stochastic?) and perpetuating mechanism(s) by which the interaction of wild-type α-syn with itself is altered, but similar to familial cases, the resulting unbalance between the different species seems to be core to the disease pathogenesis.

Importantly, regardless of being composed of mutated or wild-type protein, these fibrillar and oligomeric forms affect cellular function (usually causing dysfunction). For example, these species affect the interaction of α-syn with lipid (loss of membrane binding) and protein-binding partners [107]. As mentioned, only α-syn assemblies (oligomer, fibrils) associate NKA, disrupting its positioning in the membrane with deleterious effects [158], and whereas monomeric α-syn increases ATP synthase efficiency, oligomeric α-syn inhibits ATP synthase. The latter effect results from oxidation of the β-subunits resulting in lipid peroxidation and increasing the probability of permeability transition pore (PTP) opening, triggering mitochondrial swelling and ultimately cell death [107], and illustrates the gain of function toxicity for these supramolecular species in the mitochondria. Aggregates of α-syn accumulate within Lewy bodies (LBs). One report has claimed that LBs could be toxic by impairing cellular function through divergent mechanisms: In PD, by an interaction of α-syn with DNA to cause nuclear degradation (not clear though how cytoplasmic LBs could interact with the nuclear material), while in DLB, by drawing mitochondria into the LB compromising their integrity [141]. However, LBs could also be interpreted as markers of surviving neurons, given that they are present in the remaining neurons at post-mortem in patients with PD, or often in post-mortem tissue from asymptomatic individuals, and given that there is mixed evidence correlating severity of disease and propensity of proteinaceous inclusions [131]. In any case, what is clear is that the accumulation of aggregates into LBs [and glial cytoplasmic inclusions (GCIs)], even if not pathogenic themselves, probably reflects an inability of cells to effectively clear waste proteins due to dysfunction in clearing mechanisms (e.g., autophagy) with subsequent induction of ER and lysosomal stress [187]. The substantia nigra pars compacta (SNc) in PD brains could be a particularly vulnerable region, as there is a suggestion it shows a decrease of chaperone-mediated autophagy (CMA) activity [7], which could fail to remove pathogenic α-syn which itself could further inhibit CMA [46]. A potential compensatory upregulation of macroautophagy could be potentially also inhibited by pathogenic forms of α-syn, LRRK2 and glucocerebrosidase (GBA), as it has been shown for familial variants [6, 154, 184]. It would, therefore, be logical to deduce that those cell types and anatomical areas with more propensity to form these pernicious high molecular species (either by an increased production of the monomeric substrate, or by deficient controlling and clearance mechanisms) would be more prone to degeneration.

As already mentioned, α-syn is a widely expressed pre-synaptic protein that has been shown to be important in several aspects of neurotransmission. Therefore, it is possible that some aspects of α-synucleinopathies result from a loss of the normal function of α-syn, for example, if there was a dysregulation in the production of α-syn aggregates trapping the protein away from the conformation species needed for a particular physiological role. It has been shown that disease-causing mutations in α-syn result in an increase in the ratio of monomer:tetramer structures of α-syn, where helical tetramers of α-syn are proposed by some researchers to be the physiological form of α-syn. While the existence of α-syn tetramers has been questioned, these data would suggest that stabilising the physiological tetramers would be protective against α-synucleinopathies [52]. In cases harbouring *SNCA* pathogenic missense mutations, several mechanisms could potentially lead to a loss of function mechanism, including haploinsufficiency and epigenetic silencing [179]. A putative loss of an anti-aggregation function for wild-type α-syn was suggested in a PD patient with onset at 32 which carried the H50Q mutation in a homozygous state. The H50Q promotes aggregation ex vivo, and this is reduced when the wild type is also present. This may represent a low penetrance allele in the heterozygous state due to a putative protective role for the normal *SNCA* allele partially and variably counteracting mutations with low pathogenic potential, which could be lost in homozygous state. However, it should be noted that α-syn knockout (KO) animals do not report parkinsonian phenotypes and no known disease-causing α-syn mutations arise from mutations with a clear loss of function (e.g., leading to lack of protein expression), indicating that complete loss of function of α-syn is not associated with parkinsonian disorders. This may be due to redundancy between α, β, and γ-synuclein, as KO of all three synucleins is necessary to expose any age-dependent neuronal dysfunction [72].

Following the development of the Braak staging hypothesis for PD (Braak’s PD staging), toxic spreading of α-syn has been an interesting avenue of study, albeit a controversial one [24, 165]. As mentioned, it has been determined that α-syn can be released from both neurons and glia, and subsequently taken up by surrounding cells (both neurons and glia) [37]. α-Syn release is now known to be increased following high neuronal activity and, in this context, different α-syn species could spread at different rates in different neuronal and non-neuronal populations [108, 134, 136, 188]. Depending on intracellular or extracellular location, α-syn will differentially associate with binding partners by virtue of their expression patterns. Furthermore, α-syn may occupy different structures due to environmental factors, including pH, which differ intra- and extracellularly and affect α-syn state [59]. Extracellular α-syn taken up by microglia has been shown to initiate immune responses, which can result in neuroinflammation and may contribute to cell loss; this is especially thought to occur in MSA [175]. All these factors could contribute to the differential cellular and regional vulnerability in α-synucleinopathies.

### Innate cellular properties for vulnerability of the substantia nigra

**Fig. 2:**
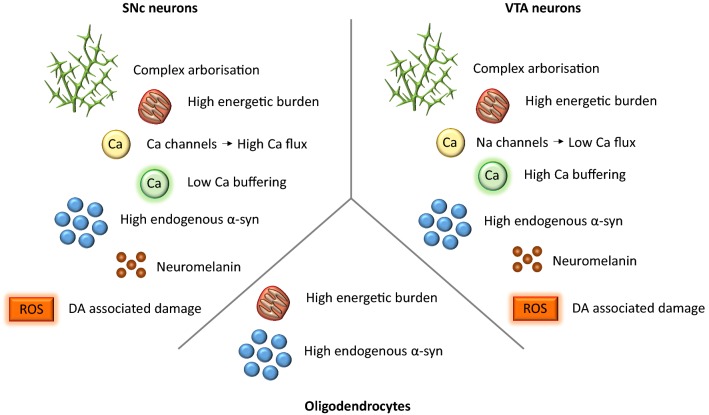
α-Syn selective vulnerability of SNc neurons/VTA neurons/oligodendrocytes. While complex arborisation, neuromelanin content, DA associated damage, high energetic burden and α-syn levels are common features of both SNc and VTA neurons, there are fundamental differences in calcium flux and buffering that may account for differential vulnerability in these cells. However, the fact remains that these properties have not been shown to be core feature of cells that degenerate in other α-synucleinopathies such as oligodendrocytes. More studies focusing on glial populations, and perhaps their calcium metabolism, are needed to determine whether this is a critical feature of α-synucleinopathies

Some of the factors which determine vulnerability to degeneration in α-synucleinopathies are best characterised in the SNc DAergic neurons. These neurons are central to these disorders as their degeneration manifests as the akinetic−rigid syndrome of PD/DLB and also of MSA. Most of the studies have focused, however, on PD. Comparing properties of vulnerable SNc DAergic neurons with their PD-resistant neighbours in the ventral tegmental area (VTA) have illustrated that metabolic, Ca^2+^ and anatomical factors are being important (Fig. 2). Here, we discuss some of the properties of SNc DAergic neurons that predispose them to degeneration and highlight factors which are thought to also apply to MSA and DLB.

#### Metabolic burden and Ca^2+^

There is an enormous metabolic burden placed on midbrain DAergic neurons, due to a number of intersecting factors, including their impressive architecture, Ca^2+^ handling capacity and DA itself being potentially toxic [138, 164]. Midbrain DAergic neurons have a tonic pacemaker activity, which following salient information switches to transient high-frequency bursting [157]. These signals need to be propagated to the striatum, where DAergic neuronal axons form enormous complex arbors of which only a fraction of potential release sites form typical synapses and DA release is under profound pre-synaptic control by other neurotransmitters [106]. Maintaining this complex architecture is thought to place a significant energetic burden on the neurons. The cholinergic projection neurons from basal forebrain, which frequently degenerate in DLB are also tonically active neurons with extensive, highly branched axonal arbors, suggesting that these factors may be shared across a proportion of the cell populations affected by α-synucleinopathies [170]. Interestingly, many of the mechanisms for metabolic strain are shared between SNc and VTA midbrain DAergic neurons, posing the question of why are SNc DAergic neurons particularly vulnerable when VTA neurons are relatively spared in PD? One aspect that differs between SNc and VTA DAergic neurons it their relationship with Ca^2+^. SNc DAergic neurons utilise the L-type calcium channel (LTCC) to support their pacemaker function, whereas VTA neurons rely on Na_v_ channels [77]. Striatal DA release in dorsal striatum innervated by SNc DA neurons is regulated by an unusually broad range of VGCC subtypes which have relatively loose spatiotemporal relationship to DA release compared to the nucleus accumbens innervated predominantly by VTA DAergic neurons [29]. In addition, VTA neurons express higher levels of the Ca^2+^-binding protein calbindin which may support intracellular Ca^2+^ buffering, and also regulate coupling between Ca^2+^ entry and downstream signalling cascades, which further divert VTA DAergic neurons from energetically demanding processes [68]. In contrast, calbindin levels are low in SNc and other nuclei affected in PD such as the locus coeruleus and the dorsal motor nucleus of the vagus, or the levels drop with age, such as in the basal forebrain [69]. Interestingly, oligodendrocytes, which are especially sensitive to degeneration in MSA, are also known to have an unusually high metabolic demand, suggesting that toxic nature of α-syn is exacerbated by high metabolic demand [26].

Cells with net elevated levels of intracellular Ca^2+^ could be prone to a vicious cycle in which high Ca^2+^ could induce α-syn to oligomerise into certain species capable of forming Ca^2+^ permeable pores [49], with further increase of Ca^2+^ influx and further oligomerisation ultimately leading to cell death.

Loss of striatal DA is central to much of the motor dysfunction in PD. However, DA itself is a potentially toxic molecule to DAergic neurons. DA is readily oxidised to form ROS compounds (DA-quinones) which have been shown to accelerate mitochondrial dysfunction leading to cell death [82]. The potential for intracellular DA to produce harmful species is dependent on several factors, particularly dopamine active transporter (DAT) activity. When DAT:VMAT (vesicular monoamine transporter) ratio is high, there is more unpackaged DA within the cytosol. α-Syn has been shown to increase DAT function, leading to DA uptake being faster [38]. One could postulate an additional vicious cycle occurring, whereby α-syn potentiates DAT function, clearing already compromised DA release faster, meaning that more DA release events are required for analogous downstream signalling, leading to compensatory upregulation of tyrosine hydroxylase (TH) leading to high intracellular DA levels resulting in more DA-ROS being created. Furthermore, α-syn increasing DAT function could increase the propensity for these neurons to take up environmental toxins including paraquat and rotenone, further indicating the potential toxic synergy between α-syn and DA biology [65]. Similar mechanisms could apply to other brain nuclei (other than the SNc and VTA) in PD and MSA-affected brains which use monoamines as a neurotransmitter (e.g., the locus coeruleus or the A2 neuronal populations of the dorsal motor nucleus of the vagus). However, many vulnerable neuronal types are not monoaminergic. To this respect, it is worth noting that α-syn also has potential to dysfunctionally interact with other neurotransmitters including glutamate, whereby α-syn can increase glutamate release and α-syn release increases with neuronal activity generating a further vicious cycle leading to glutamate-induced cytotoxicity of both surrounding neurons and glia [76]. It is unresolved if other neurotransmitters could also generate deleterious vicious cycles between α-syn and release; however, it is noteworthy that it is cerebellar glutamatergic inputs that are particularly vulnerable in MSA [101].

#### Anatomical factors and connectivity

A key factor thought to underlie specific neuronal loss in α-synucleinopathies is due to their connectivity, potentiating the spread of α-syn misfolded aggregates. Although the evidence supporting that α-syn shares some properties with prions is increasing, there is still a controversy about the prion hypothesis in α-synucleinopathies. This is discussed below, together with the possibility that some of these apparent discrepancies emerge from the methods used to visualise α-syn pathology. Nevertheless, it is possible that the spread of α-syn pathology is modulated by additional convergent factors. The local environment seems to be important. In addition to categorizing the striatum along anatomical axes, the striatum can be subdivided into two histochemically defined organizations known as striosomes (or patches) and the surrounding matrix, which are classically defined by their preferential expression of Mu-opioid receptors or calbindin-D28K, respectively. Striosomal (patch) regions within the striatum are particularly vulnerable in PD, and although they seem to receive more innervation from the ventral SNc (more vulnerable to degeneration in PD), individual DAergic neurons do not seem to selectively innervate either striosomes or matrix regions [28]. This suggests that even within a single neuron, axonal compartments are variably vulnerable, and therefore, the microenvironment is likely to be influential modifying the cellular vulnerability due to its connectivity. Interestingly, the same species of α-syn can form different pathological structures within certain cell types, illustrating the difficulty in tracking α-syn spreading pathology [136]. The extracellular microenvironment is likely to be heavily influential on glia biology which, as already mentioned, is strongly implicated in α-synucleinopathies. For example, local anatomical factors seem to explain the pattern in which astroglial cells accumulate α-syn in LB diseases paralleling the stages of intraneuronal pathology [25], although it is not clear if this potential transfer of α-syn species between neurons and astroglia is a required step in the spread of disease. Therefore, the anatomical build-up, the local microenvironment and connectivity could act by imposing permissiveness bridges or barriers in the generation and spread of self-templating species. It would not be unthinkable that the efficiency at which a cell-type transfers the pathological species may be uncoupled to their sensitivity (or resistance) to dysfunction and cell death. In this way, some highly efficient cell types in transferring these species (and particularly if anatomically strategically situated), but resistant to their noxious effects, may become gateways/motorways for disease dissemination. These factors need also to be taken into account when attempting to reconstruct the sequence of event by interpreting post-mortem tissue.

### Genetic predisposition to differential vulnerability

#### Pathogenic mutations and epigenetic modifications

In most cases with *SNCA* genetic alterations (such as the A53T, E46K, and H50Q missense mutations and the CNVs), a disease characterised by LBs was seen when neuropathology was available [139]. The pattern of distribution of LBs followed broadly that of sporadic PD cases, and this pathology also had a clinical correlate paralleling sporadic disease. In this respect, it is worth mentioning that cases harbouring the A53T mutation have prodromal symptoms including olfactory dysfunction, depression, gastrointestinal dysfunction, and rapid eye movement (REM) sleep behaviour disorder (RBD) at the same frequency or even more than in sporadic disease [94], likely reflecting a very similar LB pathology distribution. While cases with missense mutations could have variable LB extension and some mutations appear to be more aggressive than others, a clearer dose effect is found in CNVs cases, whereas although in general terms showed typical LB pathology, duplication cases showed more frequently brainstem restricted disease that correlated with an akinetic–rigid syndrome and triplication cases showed a diffuse disease that correlated with dementia. Some variability of distribution of pathology and symptoms, even within the same family, is present in both missense and CNVs’ mutations, indicating additional factors. More recently, two mutations in *SNCA*, the G51D [91] and the A53E [132] have been associated with severe and early phenotypes including both PD and MSA features, with both neuronal and glial (including oligodendroglial) α-syn inclusions and affecting neuroanatomical areas typically affected in both PD and MSA. Interestingly, CNV cases can also have MSA-like pathological features [22].

At least three conclusions can be drawn from the analysis of familial cases with *SNCA* mutations. The first is that if we accept that at least part of the pathological lesions results from the expression of the mutations within the affected cells, this provides powerful evidence supporting the idea of a partially cell-autonomous process, i.e., that differential cellular (and regional) vulnerability could emerge from expression of genetic determinants within each cell. It is, nevertheless, possible that genetically determined diseases are also susceptible to prion-like spread. If this is the case, the resulting pathology would be a combination of cell-autonomous and non-cell-autonomous processes.

The second is that if different mutations in the same gene can give rise to PD or MSA-like phenotypes, then part of the differential cellular and regional vulnerability in α-synucleinopathies may be due to different intrinsic properties of the offending α-syn, expressed at least partially in a cell-autonomous way. It remains to be seen if these missense mutations give raise to different strains in any way equivalent to their counterpart in sporadic disease, but the fact that the E46K mutation inhibits MSA α-syn replication suggests that the conformation of the PD-causing mutation E46K is different from the strain found in sporadic MSA [185, 191].

A third consequence is that it would be reasonable to consider that the level of expression of α-syn could influence differential vulnerability, as CNVs causing PD/DLB such as the duplications and triplications mentioned, have been shown to lead to higher expression of α-syn. These CNVs of *SNCA* vary greatly in size, from ~ 200 kb to > 6 Mb, and very often include other genes, although the size or additional genes do not seem to have a major influence on the phenotype [22]. It is thus the higher (but potentially variable between cells types) expression of α-syn which appeals as a potential mechanism translatable to sporadic disease.

**Fig. 3:**
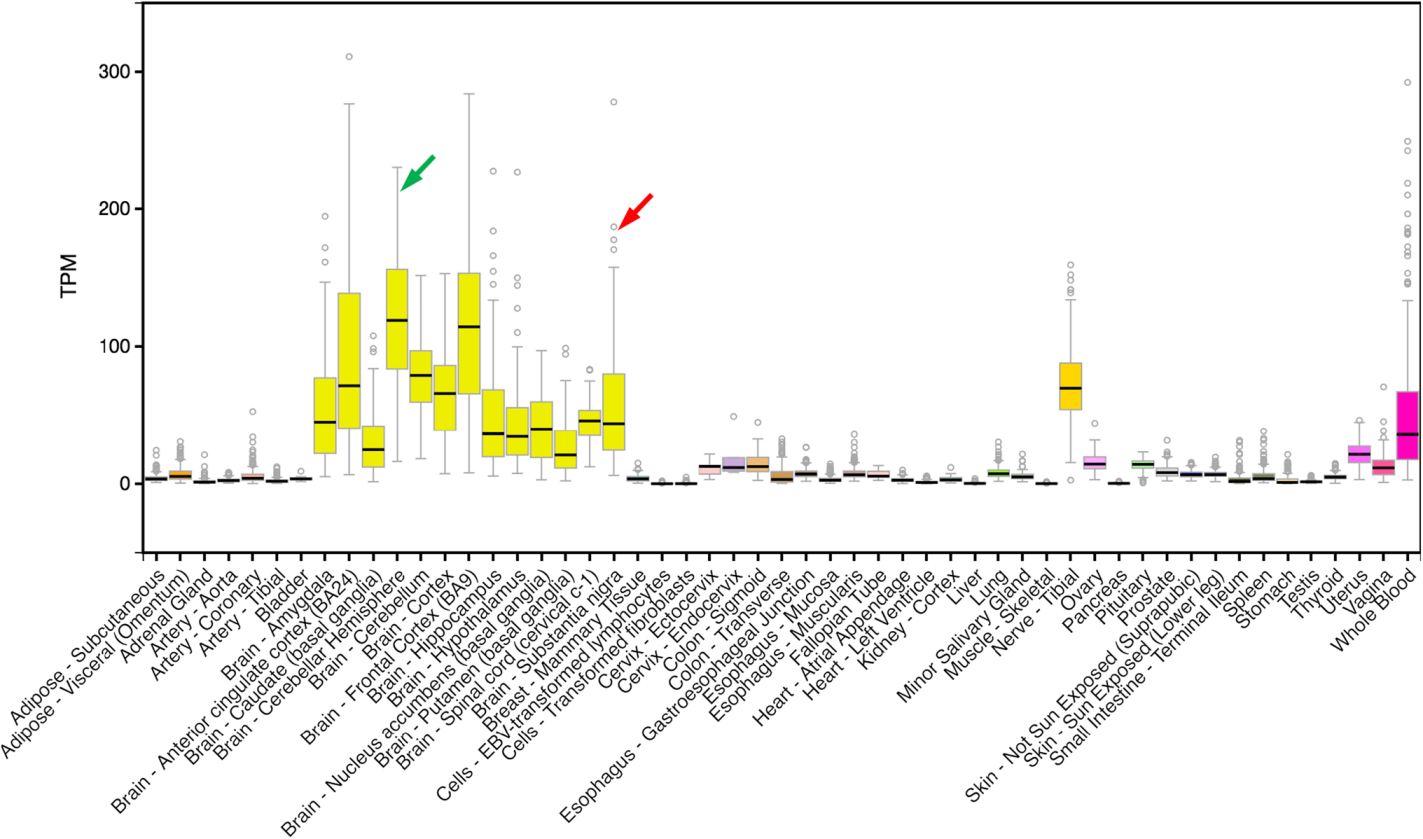
GTEX expression data. This analysis shows that the median expression of *SNCA* in the substantia nigra (red arrow) is lower than some other parts of the brain, e.g., cerebellar hemispheres (green arrow). Brain expression is overall high (light yellow plots), although high expression is also noted in tibial nerve (dark yellow plot) and whole blood (magenta). Vertical axis: transcripts per kilobase million (TPM)

Therefore, and given that *SNCA* is expressed very widely, is *SNCA* expression higher in vulnerable brain regions/cell types in sporadic disease? Review of the Genotype-Tissue Expression (GTeX) data suggests the opposite, with the median level relatively low in the substantia nigra, although there is considerable variation (Fig. 3), and the joint highest level being in cerebellar hemispheres, which are generally spared in PD [43]. Furthermore, GTEX data do not reveal any splicing expression quantitative trait loci (eQTL) in substantia nigra, which could have led to alternative potentially harmful splice variants, or any significant tissue-specific eQTL in substantia nigra driving higher expression. The picture with regard to MSA is even less clear: the primary α-syn pathology lies in oligodendrocytes, although neuronal involvement is increasingly appreciated [48]. There have been many doubts about whether α-syn was even expressed in oligodendrocytes, although increasing evidence demonstrates that it is, and possibly even at a higher level than in DAergic neurons [8, 53]. As these data are from homogenates, rather than purely DAergic neurons, reviewing data from isolated isolated human neurons may be more informative. For example, in laser-captured neurons (Suppl. Figure 1), the level of expression of α-syn in DAergic neurons has been recently shown to be actually similar to that in temporal cortex neurons, although higher than in motor cortex pyramidal neurons [56]. In some brains, the expression was highest in substantia nigra DAergic neurons, perhaps “tipping the balance” in these cases. All these differences could contribute to selective vulnerability.

An important related question to consider is: can substantia nigra DAergic neurons degenerate without α-syn pathology? If so, this would suggest that their selective vulnerability does not have to depend, at least exclusively, on α-syn. This would be the case in parkinsonian disorders such as progressive supranuclear palsy in which there is accumulation of tau, rather α-syn, in the substantia nigra. Studies on other monogenic forms of PD have also supported this [156]. Mutations in *PRKN*, which cause a recessive young onset form of PD, generally do not lead to LBs, or, if they are present, they are few and restricted [54]. The most commonly mutated gene in late-onset PD, *LRRK2*, and in particular the most frequent mutation G2019S, is generally associated with LBs, but this is not the case for all *LRRK2* mutations, with some cases showing tau pathology or even no specific inclusions [166]. The divergent pathology in cases with *LRRK2* mutations may suggest that LRRK2 lies upstream α-syn and tau in the cascade of pathogenic events [5]. While *LRRK2* mutants have been shown to exacerbate the α-syn and tau pernicious effects and *lrrk2* knockout animals exhibit protection from α-syn toxicity, it remains to be seen if *LRRK2* toxicity would be abrogated in the absence of α-syn (or tau). We do not wish to question the key importance of α-syn pathology in sporadic PD, but it clearly appears that in some genetic forms a PD phenotype with substantia nigra DAergic loss can result without LB formation. This suggests that smaller aggregates may mediate the α-syn pathogenicity, in which case the absence of α-syn (or tau) should abrogate *LRRK2* or *PRKN* mediated toxicity. If this is the case, these theoretical smaller aggregates in *LRRK2* and *PRKN* mutated cases without LBs could perhaps be demonstrated with novel techniques, such as the recently developed α-syn proximity ligation assay (AS-PLA) that we comment on below. Alternatively, the selective vulnerability in these cases may be conveyed by other factors.

Extensive GWAS have shown several single nucleotide polymorphisms (SNPs), including several within the *SNCA* locus, are associated with PD. It is reasonable to expect that only a gene expressed in a relevant tissue or cell type could exert risk, and many genes expressed in a tissue may exert their effect through regulated networks. Significant enrichment for genes highly expressed in relevant tissues and cell types was seen in several traits and illnesses [62]. A detailed study of mouse forebrain, olfactory, and midbrain neurons identified a transcriptional profile of post-mitotic SNc neurons, with two co-expressed gene modules in them associated with PD pathway KEGG gene sets [84]. PD-associated regions from GWAS [39] were significantly enriched for genes expressed in DAergic neurons, suggesting that this may play a role in their vulnerability. Intriguingly, *LRRK2*, was not expressed in DAergic neurons in this study, leading to the suggestion that it may act through microglia. This difference could help explain the divergent pathology discussed above. Other studies, however, have shown *LRRK2* expression in substantia nigra DAergic neurons from mouse and human [5, 161]. The way in which *SNCA* SNPs modulate risk is complex, with at least three independent signals, and a different pattern in PD and DLB shows that the effect of each independent signal is highly phenotype specific [137]. The strongest signal in PD is absent in DLB, while the secondary signal is significant in both. The risk alleles may affect transcription sites, leading to different relative abundance of transcripts with varying lengths of untranslated regions, and this effect could be tissue or cell-type specific.

The levels of *SNCA* expression could also be modified by epigenetic mechanisms such as DNA methylation. Studies on DNA methylation could, therefore, contribute to revealing tissue- or cell-specific *SNCA* expression regulation. The conflicting studies on this topic have been reviewed [133]. One interesting study showed concordant changes in blood and frontal cortex, but the substantia nigra was not studied [111]. Methylation in the substantia nigra was decreased in PD in another study [113], but no cell-type specific data in health or disease are available yet to our knowledge. There are major technical developments in understanding chromatin accessibility at the single cell level, including in the human and mouse brain [47, 97]. The substantia nigra has not been investigated in these studies, but interestingly enrichment for Alzheimer’s disease (AD)-associated SNPs was observed in both in microglia, supporting their key role in that disease. Dissecting the transcriptome of each cell type is expected to advance rapidly with more data from the GTEX and the Human Cell Atlas project [148], to help address these questions in a more definitive way.

#### Genome mosaicism

##### Nuclear genome

The nuclear genome of all cells in the body was considered to be identical and static, apart from cancer and some rare disorders. The topic of brain mosaicism, the presence of cells with different genomes due to post-zygotic mutations, and a possible role in brain disorders, is now generating a lot of interest [114]. Recent evidence demonstrates multiple recombination events in the *APP* gene occurring in somatic neurons not only in AD cases but also in normal neurons, in a process that seems to involve age-dependent neuronal “retro-insertion” of RNA [100]. Could there be somatic genomic changes in DAergic neurons which make them vulnerable? It is not clear if DAergic neurons may be particularly prone to somatic mutations, and if so, why this would be, and what sort of mutations would arise preferentially. There have already been some studies addressing this question. One study reported mosaic aneuploidy (gains for both chromosomes studied) in PD substantia nigra neuromelanin-positive neurons and proposed aberrant DNA synthesis leading to whole genome duplication in these [83]. Another study assessed total DNA content in substantia nigra and other neurons; in LB diseases, several cell types, including substantia nigra pigmented neurons, had higher DNA content than controls, but the type of additional DNA was not clear [189]. A recent targeted study of the *SNCA* gene using fluorescence in situ hybridization (FISH) showed low levels of CNVs (gains) in DAergic neurons (distinguished by neuromelanin) in most PD and MSA cases studied (31/40 and 4/5, respectively) [120]. This phenomenon was seen less frequently in controls (10/25), and at a significantly lower overall level, and one of these controls did actually have LB pathology on further review. These gains were less often seen in non-pigmented cells. Further work is needed to determine the extent to which this is peculiar to the substantia nigra DAergic neurons, and whether it reflects a tendency towards more general genomic disturbance. It is also not clear if these may have arisen during embryogenesis rather than post-mitotically, as recent work suggest CNVs are more common in younger controls, consistent with an early origin and selective vulnerability to aging [42].

##### Mitochondrial genome

In contrast to the nuclear genome, the mitochondrial genome has long been known to be variable, with heteroplasmy well established. The evidence for a role in PD through preferential acquired mutations in DAergic neurons has been somewhat contradictory, with early reports of deletions being more common in PD. The most recent evidence does suggest an excess of deletions, but not missense mutations, as well as a failure to increase mitochondrial DNA (mtDNA) copy number with age in PD, unlike controls [55]. Furthermore, pathway analysis of exome data has shown an enrichment of rare inherited variation in the pathway controlling mitochondrial DNA replication and repair, indirectly supporting this as a mechanism contributing to disease rather than a result of damage from oxidative stress [67]. How do mtDNA mutations in DAergic neurons contribute to PD? Mitochondrial respiratory chain dysfunction was reported for complex I in the substantia nigra 20 years ago [74]. Later, single neuron studies showed that complex I and II are the main affected parts of the respiratory chain, with complex IV involvement possibly later or secondary [73]. Although the assumption has always been that the complex I deficiency is limited to the substantia nigra, and of clear pathogenic relevance, this has been challenged by the recent discovery of complex I deficiency in several brain regions in PD, when laser capture was used, including spared areas from pathology like the cerebellum. Furthermore, the complex I deficiency did not correlate with mtDNA damage in areas outside the substantia nigra [64].

Is there something unique about substantia nigra DAergic neurons which leads to DNA damage and secondary dysfunction, in the mitochondrial or even nuclear genome? α-Syn has been shown in animal and cell models to trigger the DNA damage response, possibly leading to single and double strand breaks [119]. This is mediated at least partly by reactive oxygen species, and specific to DAergic neurons, presumably because of the excess ROS generated by DA metabolism and the factors discussed above.

## Strains and phenotypic diversity

Although, α-synucleinopathies are defined by the presence of α-syn inclusions, the exact nature of the inclusions is specific to each disease. The variability in these gross structures may be linked to different upstream conformational assemblies that dictate the phenotypic diversity and regional vulnerability ultimately resulting in the stereotypical manifestations of each disease.

### The diversity of α-syn inclusions

LBs, also known as neuronal α-syn inclusions, are the pathological hallmark of PD and DLB. The structural properties of LBs localized in the brainstem or cortices differ. Brainstem LBs are more compact, with α-syn fibrils more orderly arranged compared to cortical LBs [90, 95]. In MSA, GCIs containing α-syn are present in oligodendrocytes. As said, the basis for GCIs had been somewhat mysterious as oligodendrocytes were thought until recently to express little or no α-syn. GCIs have been shown to contain even more compacted α-syn than LBs [136], and to be made up of an annular α-syn structure [140]. In addition to the biochemical and biophysical studies that have shown, these differences in structure, distinct properties of brainstem and cortical LBs, and GCIs, have been observed by staining with antibodies specific for particular α-syn three-dimensional conformations. For example, one study used the monoclonal antibody Syn7015, that seemed to be selective for a particular recombinant α-syn aggregate, and showed that GCIs were preferentially detected [136], perhaps suggesting inclusions were made preferentially of different conformational strains. Recently, the AS-PLA, a method that allows visualisation of α-syn oligomers in situ with preservation of the cellular detail, has been developed [150]. Using the AS-PLA technique, it was observed that brainstem LBs and GCIs were unstained or had punctate staining on their periphery, consistent with their compacted fibrillar structure [150]. The crown of oligomers around the compact inclusions was postulated to reflect either recruitment of oligomers into the inclusions or perhaps release of oligomers from them. In contrast, cortical LBs displayed strong AS-PLA staining, highlighting their distinct structure. As mentioned, it will be interesting to see if this and other techniques can demonstrate α-syn aggregates in *LRRK2* and *PRKN* mutated cases without LBs.

Not only do the inclusions seem to have distinctive features, but in general terms, their distribution throughout the brain would appear to also follow at least partially different patterns. In MSA, GCIs are distributed in the olivopontocerebellar system, the nigrostriatal system and the autonomic system [162]. PD and DLB are both defined by the presence of LBs in the brainstem, limbic and cortical regions (the predominant pathology in DLB is in limbic and cortical areas, while in PD brainstem pathology dominates).

### α-Synuclein strains and the prion-like hypothesis

#### The prion-like hypothesis of α-syn

Based on neuropathological studies, staging systems for the progression of α-syn pathology in PD and DLB were proposed [12, 24]. The stereotypical progression of α-syn pathology suggested the possibility for a spreading of misfolded α-syn along anatomical pathways. This suggestion found support in the work of two laboratories which revealed that pathological α-syn was detected in healthy DA neurons that were transplanted in some PD patients for the treatment of the akinetic–rigid symptoms, suggesting prion-like properties for α-syn [70]. These are powerful observations, but we must note that the conclusion that these results demonstrate propagation of pathology in humans has been challenged [60] and alternative mechanisms such as involvement of inflammatory responses have been alleged. It has been argued that pathology only arose in cases in which small cellular aggregate suspensions or small tissue pieces, which elicited extensive microglial activation, were transplanted, while a similar number of cases of PD patients with transplants of dissociated neurons, which did not elicit microglial response, were perfectly healthy after an equivalent number of years [118].

Since then, mounting evidence from cell and animal models supports the hypothesis that disease can spread through the brain by self-propagating α-syn aggregates with templating ability. In vitro, over-expressed α-syn in SH-SY5Y cells was able to transmit to naive SH-SY5Y cells and induce inclusion formation [51]. α-Syn pre-formed fibrils (PFFs, recombinant α-syn aggregates) were able to seed new aggregation in HEK293 cells overexpressing α-syn or in primary neurons expressing endogenous levels of α-syn [109, 178]. Injection of PFFs or lysate from the brains of DLB patients into mouse brain led to the development α-syn pathology at the injection site, which spread to neuroanatomically connected regions [108, 112]. Injection of LB material into the striatum of non-human primates resulted in α-syn positive staining in the substantia nigra, globus pallidus, precentral and superior frontal gyrus and temporal cortex, supporting the ability of abnormal α-syn to propagate to distant brain regions and to trigger neurodegeneration [147]. In another experiment, researchers performed an intracerebral injection of α-syn in mouse brains before and after callosotomy to dissociate the two brain hemispheres. Results showed that the seeding ability of α-syn appeared to be reduced in the contralateral hemisphere in the mice that had received callosotomy before α-syn inoculation. On the other hand, in the mice receiving callosotomy after α-syn inoculation, the pathological protein was found to have been transmitted and accumulated to the contralateral hemisphere. Callosotomy blocked an important route for α-syn propagation through the corpus callosum; however, this was not completely abolished as α-syn might still propagate through limbic routes, including hippocampal traffic and the anterior commissure [127].

As mentioned, although the evidence supporting the hypothesis that α-syn shares properties with prions is increasing, the applicability of this hypothesis in α-synucleinopathies remains controversial. For example, there is a significant portion of post-mortem samples that are not explained by Braak’s PD staging and it is also evident that not all cells within a neuroanatomical nucleus are equally affected [165]. There is evidence that accumulation of α-syn aggregates in the peripheral nervous system, and particularly in the peripheral autonomic nervous system, precede brain pathology. But why would α-syn spread initiate from the periphery when it is most strongly expressed in the brain? It has been argued that peripheral parasympathetic neuronal terminals in the gut and olfactory sensory neurons in the nose may be exposed to a noxious agent, triggering local pathology and subsequent spread. However, no clear exposure route to noxious agents is evident for sympathetic nerves and ganglia consistently affected early in PD such as the cardiac sympathetic nerve [128], while these neurons do share cell-autonomous features with other affected areas, such as the production of catecholamines. Importantly, no cases with α-syn peripheral nervous system pathology without the brain also being affected have been found in 600 whole-body comprehensive autopsies [105]. More intriguingly, α-syn aggregates from post-mortem PD stellate sympathetic ganglia failed to promote α-syn pathology in the brain of inoculated mice [146]. Other observations that challenge that the propagation of α-syn pathology emerge from its prion-like properties include that the severity of clinical progression does not fully correlate with LB pathology progression and that the strength of connectivity between brain areas does not correlate with spreading patterns of α-syn [165]. It is possible that some of the gaps in this theory reflect the fact that current α-syn immunohistochemical methods used so far label late-stage compact aggregates such as LBs, Lewy neurites (and GCIs). The novel AS-PLA method allowed the authors to identify early aggregation stages within cells and a striking previously unrecognised neuropil (presumably axonal) oligomeric pathology in anatomical areas not yet affected by LB pathology [150]. It is possible that the use of this and similar methods for specific protein species may lead to a better definition of the pathological routes of α-syn propagation, perhaps solving some of these apparent discrepancies (although surely eliciting other questions).

#### α-Synuclein strains

In classical prion diseases, a key feature in addition to the templating ability of the pathological protein, is the presence of a variety of strains. The term strain has been used to denote distinct pathological aggregates of the prion protein that have varying abilities to seed and propagate, and with different propensities for seeding aggregation in particular brain regions. Intense investigation is now focusing on whether different α-syn strains exist.

The well-known propensity of α-syn to aggregate has been exploited to generate aggregated forms of recombinant α-syn protein in vitro. Using this experimental setup, it has been well demonstrated that parameters such as salt concentration, temperature, cross-linking agents and physical force by shaking leads to the formation of α-syn aggregates that display distinct structural, biophysical and biochemical properties [23, 44, 49, 122]. One study demonstrated that varying salt concentrations led to the formation of two structurally distinct aggregates, which they termed fibrils and ribbons. Importantly, both structures were able to imprint their structural conformation onto naïve α-syn molecules, a key feature of pathological prion proteins [23].

On the other hand, repeated rounds of seeding of α-syn PFFs have been shown to lead to the formation of a strain that is distinct from de novo PFFs [75]. Interestingly, the strain that resulted from multiple rounds of seeding was less efficient at seeding new α-syn aggregation but was particularly potent at seeding tau aggregation in vitro and in an animal model. Given the hypothesis that PD/DLB pathology begins in the brainstem and progresses through the brain to the cortex, it is possible that repeated rounds of seeding as the aggregates move through the brain modifies the pathological form of α-syn. This may have implications for the observed differential structures of brainstem and cortical LBs and for the presence of tau inclusions in cortex of patients with PD and DLB, particularly in those with dementia.

The studies that utilize in vitro formed aggregates provide a proof of principle that α-syn aggregates are capable of self-propagating and to form different species (and presumably strains), but how these forms relate to aggregates present in diseased brain is poorly understood. The demonstration in multiple studies that brain extracts from patients with α-synucleinopathies were sufficient to induce the presence and spread of α-syn pathology in animal models provides evidence for the presence of self-templating α-syn in diseased brain [112, 143, 183]. They are also starting to show differences which could be attributed to different strains. In one study, brain extracts from MSA patients, but not healthy controls or PD patients, led to neurodegeneration in a particular type of α-syn transgenic mice, accompanied by α-syn inclusions [143]. Similarly, a recent study proved the different spreading capacity of α-syn extracts from MSA and DLB patients. Three months after the inoculation of the extracts in the striatum of the C57BL/6 mice, the levels of MSA-α-syn were enhanced in brain areas including the striatum, frontal cortex, amygdala, and substantia nigra, while DLB-α-syn induced low levels of Lewy-neurite-like α-syn pathology. The aforementioned findings suggest that unique strains of α-syn aggregates are responsible for each disease. The exact biochemical differences in the α-syn seeds present in MSA or PD brains, and how they relate to their ability to preferentially affect certain brain regions or cell types, remain unknown. Nevertheless, these properties are strongly suspected to underlie part of the differential cellular and regional vulnerability in α-synucleinopathies, while at the same time different cell types and regions could potentially generate or transfer different strains with different efficiency.

Several studies have begun to address this question, for example, by injecting two α-syn strains (fibrils or ribbons) into rat substantia nigra [23, 135]. Both fibrils and ribbons were able to induce α-syn pathology in neurons, but only ribbons could seed aggregation in oligodendrocytes. This suggests there is a specific conformational feature of the ribbon conferring its ability to seed α-syn aggregation in oligodendrocytes. Conversely, the cellular environment has been shown to influence the strain of α-syn formed. GCI-derived α-syn has been shown to be approximately 1000-fold more potent at seeding new inclusions. Interestingly, α-syn PFFs seeded in the presence of rat primary oligodendrocyte lysate were able to induce significantly more pathology in a cellular model compared with the PFFs generated in rat primary neuronal lysate [136]. This suggests the oligodendrocyte cellular milieu may promote exceptionally aggressive self-templating forms of α-syn. The specific factors mediating such profound differences in the strain formed are as yet unknown, but interaction studies with PFFs and cell lysates or proteomics to identify enriched proteins in α-syn infected oligodendrocytes versus neurons could help to shed some light on this. Equally important would be to fully resolve what are the endogenous expression levels of α-syn in oligodendrocytes, as low levels could impact their ability to seed aggregation. Recent evidence from primary rat oligodendrocytes suggests that α-syn PFFs triggered the aggregation of the endogenous α-syn, which could then not be processed by autophagic machinery and led to the ultimate increase of α-syn levels and aggregation within the cell [89]. Finally, it is interesting to note that sonication is necessary in order for PFFs to be active in the brain or in culture, which inevitably questions the strain itself; are PFFs the only strain present in these preparations and, therefore, the main drivers of pathology in these experimental paradigms or is there also enrichment in other species like oligomers that should be considered as well?

As evidence continues to accumulate for the contribution of distinct α-syn strains to disease-specific phenotypes, it will be important to assess the features of strains derived from patient brain. As only small amounts of pathological α-syn can be obtained from patient brain, techniques that allow the amplification of strains of misfolded proteins such as protein misfolding cyclic amplification (PMCA) or real-time quaking-induced conversion (RT-QuIC) will become crucial to allow their properties to be studied in more detail. Furthermore, if a therapeutic strategy is intended to target α-syn aggregates to prevent their spread, it will be important to identify the primary strains, particularly as they may be modified as the disease progresses. The convergence of biomarkers, cohorts of patients at the early stage of the disease and efforts to pin down the molecular structures will allow these questions to be addressed. We believe that understanding the properties of different α-syn strains is key. The fact that different strains of α-syn are associated with particular α-synucleinopathies and may even vary throughout disease progression could also reflect the ability for different structures of α-syn to interact with specific biological partners [59, 173]. Therefore, understanding these interactions will also potentially allow us to develop novel biomarkers for α-synucleinopathies throughout their progression, which is essential for clinical trials (cohort selection and effectiveness measures), and the development of new therapeutics based on sound scientific rationale.

## Exogenous and environmental factors modulating selective regional vulnerability

We have discussed so far how the interplay of intrinsic cellular and anatomical factors of the affected populations, including their genetic background, together with different α-syn strains with capacity for self-templating are thought to explain the pathogenesis of α-synucleinopathies and their phenotypic diversity. However, exogenous and environmental factors such as neurotoxins, heavy metals, brain trauma and sleep disorders have also been implicated in neurodegenerative α-synucleinopathies [41]. We will discuss some of these factors and how they could contribute to the selective cellular and regional vulnerability in α-synucleinopathies.

### Neurotoxins

Several studies support that treatment with neurotoxins inhibiting the mitochondrial complex I and causing oxidative stress like MPTP, paraquat, and rotenone enhance the risk of developing α-syn pathology.

#### MPTP

The injection of 1-methyl-4-phenyl-1,2,3,6-tetrahydropyridine (MPTP) induces a rapid PD-like syndrome. MPTP can be accidentally synthesized when attempting to produce the opioid analgesic drug desmethylprodine, also called 1-methyl-4-phenyl-4-propionoxypiperidine (MPPP). The neurotoxicity derived after the injection of MPTP was discovered in a patient in 1976 and a further four patients in 1983 that had illegally used MPPP contaminated with MPTP. Although MPTP itself is not toxic, it readily crosses the blood–brain barrier and is subsequently metabolised to the toxic 1-methyl-4-phenylpyridinium (MPP^+^) which is uptaken by DA transporters into the neurons. By accumulating into mitochondria, MPP^+^ inhibits complex I leading to energy failure and oxidative stress [145]. As mentioned, the highly elongated and branched axons of the DAergic neurons in substantia nigra require high levels of energy that can be barely provided by the mitochondria. Therefore, the morphology and the increased energy demands of nigrostriatal DAergic neurons make them more vulnerable to MPTP compared to the DAergic neurons in the VTA [155]. It is clear from the early reported cases of parkinsonism due to MPTP that transient exposure to MPTP results in reduction in nigrostriatal DAergic function [10]. Researchers have also revealed upregulation of α-syn in DAergic neurons after administration of MPTP [176]. Although MPTP triggers α-syn accumulation, the extensive deposition of the LBs and neurites observed in PD cases, is not observed in MPTP models, even after a decade administration of MPTP [78]. Instead of LBs, α-syn amorphous bundles are observed [115]. This finding indicates that oxidative stress, inflammation, and cytotoxicity are not the only mechanisms contributing to LBs formation. Other mechanisms must also contribute to the protein aggregation in LBs [78]. Brain analysis of MPTP-lesioned non-human primates displayed a significant escalation in α-syn mRNA levels after 1 week or 1 month of MPTP treatment, with the protein being accumulated in the cell bodies of DAergic neurons [144]. Other studies support that overexpression of α-syn in mice enhances the vulnerability of substantia nigra DAergic neurons, while deletion of the protein in α-syn null mice reduces MPTP toxicity [50]. These studies indicate the participation of α-syn to pathways essential for DAergic neuron viability and alterations in the expression or mutations of *SNCA* can modulate the vulnerability of these neurons to a specific neurotoxin. Indeed, misfolded α-syn can induce activation of brain immune cells, enhanced inflammation and production of ROS making the DAergic neurons more susceptible to MPTP [99]. While accumulation of α-syn in the glial cells has been described in α-synucleinopathies [169, 180], α-syn accumulation was not detected neither in microglia nor astrocytes in a study with a primate MPTP model [115].

#### Paraquat

Paraquat (*N*,*N*′-dimethyl-4,4′-bipyridinium dichloride) is a toxic herbicide which increases cytoplasmic oxidative stress and inflammation in the midbrain leading to the selective degeneration of substantia nigra DAergic neurons [116]. Paraquat enhances the production of DA, resulting in the formation of toxic quinoproteins that affect the normal function of numerous proteins, including α-syn, and lead to cell death, explaining the specificity of paraquat for DAergic neurons [17]. Treatment of A53T mutant human α-syn transgenic mice (TgM83) with both paraquat and maneb, an inhibitor of complex III, but not with each substance alone, enhanced also the formation of α-syn inclusions in brainstem, striatum, and thalamus [125]. The temporal expression of the pathological protein is also affected by this herbicide [123]. According to Braak’s theory, α-syn distribution initiates in body areas other than the brain, like enteric nervous system (ENS) and olfactory bulb [13]. In a recent study, researchers proved that oral administration of paraquat in TgM83 mice led to an earlier expression of the pathological α-syn in the ENS, while this precocious expression was not observed in the brain of the mice [123]. At this point, it is essential to mention that the exposure to paraquat of non-transgenic or transgenic mice expressing the wild-type human α-syn did not induce α-syn pathology, underlying that in this case, the interaction between the genetic and environmental factors is essential for the progression of the α-synucleinopathy [123, 125].

#### Rotenone

Another identified pesticide that is associated with α-syn pathology is rotenone, also known for inhibiting mitochondrial complex I and producing ROS, in all cell types [104]. However, DAergic neurons, appeared to be highly affected compared to other types of neurons due to the rotenone-induced oxidation of DA [149]. The increased production of ROS as well as the anatomical features of the substantia nigra DAergic neurons explain the selective vulnerability of this cell type to rotenone [21]. In a process with parallelisms with MPTP toxicity, it has been shown that rotenone exposure upregulates α-syn and triggers α-syn oligomerization [190]. However, and in contrast with MTPT, a recent study supported that unilateral intracranial infusion of rotenone in the medial forebrain bundle and substantia nigra in mouse brain increase dramatically the bilateral aggregation of α-syn in substantia nigra, but not in the striatum, causing substantia nigra degeneration and motor disabilities [35]. Another study showed that rotenone induced the formation of eosinophilic inclusions similar to the pale body precursors of LBs [16], while western blotting analysis of rotenone-induced α-syn revealed α-syn forms with high molecular weight corresponding to dimers, trimers, and post-translational modified proteins [71]. Finally, the spreading capacity of these rotenone-induced α-syn forms between neurons was investigated by another group, where researchers using primary neuronal cell cultures proved that rotenone can initiate the progression of α-syn pathology through transneuronal and retrograde axonal transport by increasing the number of exosomes participating to α-syn release [130]. Both MPTP and rotenone share a mechanism of being mitochondrial poisons and that both increase expression of α-syn. However, there seem to be crucial differences, with rotenone, but not MPTP, being able to form inclusions more similar to PD and to induce the spread of pathology. It is possible that the differences relate to intrinsic chemical/toxicological properties of these substances. However, given that MPTP acts largely exclusively in DAergic cells, it is possible that inclusion formation and spread require not only DAergic cell-autonomous factors but assistance from other non-cell-autonomous factors generated from the other neuronal and/or glial cell types. A differential concourse of these factors may also lead to differential cellular and regional vulnerability.

### Heavy metals

Another important environmental factor that is highly associated with α-syn pathology is the exposure to heavy metals like copper, manganese, and iron.

#### Iron

Iron is a well-described metal linked to α-synucleinopathies, with numerous studies suggesting an iron concentration increased in brain areas like the substantia nigra, putamen and globus pallidus in patients with PD, MSA, and DLB [88]. Iron has a synergistic interaction with α-syn. On the one hand, intracellular α-syn facilitate the accumulation of intraneuronal iron, while on the other hand, iron promote the oligomerisation of α-syn. More specifically, the Fe(II) ion commits N-terminally acetylated α-syn, believed to be a physiologically relevant form in humans more resistant to oligomerisation [31], to a PD-relevant oligomeric assembly with a right-twisted antiparallel β-sheet component [2]. One study showed that iron induces reduction of the levels of autophagic related proteins in primary neurons and SH-SY5Y cells impairing the autophagy system and leading to the accumulation of α-syn [181]. Focusing also in the lysosomal–autophagic system, a study proved that iron-induced activation of Akt/mTOR axis in mouse midbrain substantia nigra cells expressing α-syn may be involved in preventing transcription factor EB (TFEB) nuclear translocation, inhibiting in this way the autophagosome–lysosome fusion and triggering α-syn aggregation and transmission to the neighbour cells [186]. While a correlation between iron-overloaded disorders and parkinsonism has been reported [124], other study showed that patients with iron-overload disorder do not have an increased susceptibility to parkinsonism [1]. In contrast, neurodegeneration with brain iron accumulation, a group of inherited neurologic disorders in which iron accumulates in the basal ganglia, show widespread α-syn LB (and tau) accumulation, particularly those harbouring *PLA2G6* mutations [129].

#### Copper

Copper is an element that plays a pivotal role in a number of mechanisms including the synthesis of neurotransmitters, oxygen consumption and cell signalling [41]. Both oxidative states of copper can bind α-syn [172] and copper exposure appears to accelerate the progression of α-syn pathology [66] through at least two possible mechanisms. In the first one, the interaction of copper with α-syn affects its helical conformation leading to its oligomerisation, while in the second one, copper induces the oxidation of α-syn causing its accumulation [36]. However, a protective role for copper has also been proposed. It has been suggested that the presence of copper may induce the formation of less-damaging structurally different α-syn strains showing greater ability to form less cytotoxic intracellular inclusions. These experiments would strengthen the opinion that LBs may be protective bystanders and that α-syn oligomers are the toxic species [177]. Interestingly, the H50Q variant affects copper binding [142], although as discussed, its pathogenicity has been questioned. Copper-related disorders like Wilson’s disease can present as a parkinsonism with dystonic tremor but, in contrast to disorders with iron accumulation, no LBs or α-syn accumulation is seen, suggesting that copper accumulation in these cases may not act through an α-syn-related mechanism.

#### Manganese

Another metal related to α-synucleinopathies is manganese. Manganese homeostasis is vital for energy metabolism, enzymes function, and immune reaction [40]. Chronic exposure to manganese has multiple neurological effects including the increased susceptibility of α-syn to form inclusions [79]. Manganese accumulates mainly in basal ganglia and mostly in globus pallidus, increasing in this way the vulnerability of the cells in this region [96] and manganese exposure resulted in α-syn accumulation triggered by ER stress in mouse neuroblastoma cells expressing human α-syn [9].

### Traumatic brain injury

Neuronal dysfunction and cell death induced by acute conditions like traumatic brain injury (TBI) seem mediated by enhanced inflammation, mitochondrial dysfunction, oxidative and ER stress, pathological mechanisms that have a crucial role in the development and progression of α-synucleinopathies [4]. Post-mortem analysis of human brain following TBI has revealed positive staining for α-syn, highly associated with axonal pathology [171]. In a recent study to investigate the link between TBI and neurodegenerative disorders, 5–6-week-old mice were exposed to multiple condition head impacts and the levels of several proteins were examined. The results showed that after repetitive TBI, there was an increase in brain amyloid β, tau, and α-syn in a dose-dependent manner [102]. α-Syn accumulation has also been detected in glial cells [3], predominantly in microglia compared to astrocytes [85]. Microglia probably serves the double role of uptaking α-syn for clearance and to initiate an immune response by acting as an antigen presenting cell [61]. Consistently, a previous research proved that chronic TBI rats presented overexpression of α-syn in the ipsilateral rather in the contralateral substantia nigra 60 day post-TBI. This increased accumulation of α-syn was correlated with enhanced activation of microglial cells around the area of the injury [3]. Chronic activation of microglia and other brain immune cells results in chronic neuroinflammation through the production of pro- and anti-inflammatory mediators and reactive species leading to intraneuronal oligomerisation of α-syn [121]. α-Syn fibrils, in turn, enhance the recruitment of brain immune cells to the injured area of the brain creating a vicious cycle and promoting neurodegeneration [58, 151]. Therefore, the injured brain regions appeared to be more vulnerable in developing α-syn pathology.

### Circadian rhythm alterations

An association between circadian rhythm alterations and some neurodegenerative diseases has been reported. This association could be the consequence of the spread of disease to involve the neuroanatomical substrates of circadian rhythm regulation. Alternatively, or in addition to this, it is also possible that alterations in the circadian rhythm could modify brain function and make it more susceptible to neurodegeneration, for example by altering protein homoeostasis and immune function. If that was the case, restoration of sleep patterns could have a role as a preventive or therapeutic intervention. Altered circadian rhythms have been found to be associated with a higher risk for mild cognitive impairment (which often preludes the development of AD) or even clinical dementia [168]. Altered circadian rhythms are also found in α-synucleinopathies. RDB is a sleep alteration taking place during the REM phase of sleep, the phase in which the most vivid dreams occur. This phase is normally characterised by muscular atonia. In RDB there is a loss of muscle atonia resulting in patients acting out their (frequently unpleasant) dreams with complex behaviours. A large proportion of patients with RBD evolve into a clinical α-synucleinopathy after 5 to 14–16 years [86]. Interestingly, from this common initial manifestation the patient can develop either PD, MSA or DLB. The anatomical substrate of RBD is not completely clear. Candidates include dorsal midbrain and pons [19], particularly the peri-locus coeruleus and sublaterodorsal nucleus (SLD) [20]. These are either sites of accumulation of α-syn in brain regions at early Braak’s PD stages or sites connected to them. For example, glutamatergic neurons of the SLD possess descending projections into the medulla, one of the earliest affected brain regions in LB disorders [19]. Importantly, autopsy studies have confirmed that RBD cases display α-syn deposition in the olfactory bulb, brainstem, and amygdala (all areas frequently affected in LB disorders) before converting into clinical PD or MSA [86]. The function of neurons at these sites is modified by circadian rhythm mechanisms (for example daily rhythms in circadian clock gene expression), and therefore, it is possible that these mechanisms may impinge upon physiological and also pathological elements that could influence pro-neurodegenerative aggregation [80]. As mentioned, disentangling cause and effect is even more challenging in these cases. However, and given that this could be potential therapeutic target, we think more studies are warranted to clarify if sleep disturbances could be causing or contributing to neurodegeneration. For example, if certain types of sleep disturbance or even near-normal routines/patterns of sleep (or frequent sleep deprivations) during life could influence the process of disease generation and spread through conveying differential cellular and regional vulnerability, or if, alternatively, a healthy sleep pattern may have a neuroprotective role.

### Microbiota, infections and dietary factors

The possibility that microorganisms could be causing or triggering PD arose after the 1920–1930s influenza epidemic, which was associated with encephalitis lethargica, a disease that resulted in a PD-like akinetic–rigid syndrome [117]. However, no definite proof for this or any other virus has been found in human brain so far as causatives of α-synucleinopathies. After decades in which new viruses were being postulated but never proven, the hypothesis that an environmental agent such as a virus, bacteria or misfolded protein was triggering the disease re-emerged with the Braak hypothesis, who postulated that such an agent could be entering through the nose or the gut [81]. Vertebrate food products have been suggested, but not yet proven, as a potential trigger of α-syn pathology through the ingestion of a misfolded α-syn inoculum from animal source [92]. Recent evidence suggests that gut microbes promote the motor dysfunction and brain pathology mediated by α-syn [153]. In this case, modulation of neuroinflammation, rather than a direct mechanism, has been suggested. While the importance of these hypotheses for the development and progression of the human disease remains to be fully determined, the different route of entry for these agents (e.g., nose or gut) and the mechanisms proposed (e.g., direct entry versus gut–brain neuroinflammatory axis) add more putative mechanisms to explain the differential vulnerability and resulting phenotypic variability of these disorders.

## Conclusion

It is clear that α-syn has the central role in the group of disorders referred to as α-synucleinopathies. We are just starting to understand how the combination of the niche physiology of highly specialised neurons and glia, and the extensive scope of α-syn biology gives rise to an intersecting web of factors that determine α-synucleinopathies aetiology and progression. We believe that there are several key developments that could unlock our understanding of the differential cellular and regional vulnerability in α-synucleinopathies and ultimately open the route to disease-changing treatments. These include, for example, the ability to specifically visualise and measure robustly the different endogenous types of α-syn supramolecular arrangements (conformational variants, species, strains) in biological samples from early time points in the disease, coupled with the development of non-invasive, e.g., imaging-based tests, capable of correlating with these biological measurements. While these tools could help resolve temporal and spatial pathophysiological factors, we also believe that an increase in innovative human post-mortem studies is also essential in ultimately proving novel hypotheses. As for now, we can only speculate that the disruption of the cellular metabolic state, α-syn structural equilibrium, and anatomical connectivity appears to initiate cascades of pathological processes triggered by genetic, environmental or stochastic events that result in the “death by a thousand cuts” profile of α-synucleinopathies.

## Electronic supplementary material

Below is the link to the electronic supplementary material.

Suppl. Figure 1. BRAINcode Project expression data of *SNCA* in human laser-captured cells. This analysis shows the expression of *SNCA* in the substantia nigra DAergic neurons and other cells. SNDA: substantia nigra DAergic neurons, MCPY: motor cortex pyramidal neurons, TCPY: temporal cortex pyramidal neurons, PBMC: peripheral blood mononuclear cells, FB: fibroblasts. (PPTX 160 kb)
