# Supplementary figures and images for: Selective vulnerability in α-synucleinopathies
Source: Acta Neuropathol. 2019 Apr 20;138(5):681–704. doi: 10.1007/s00401-019-02010-2 (PMC6800835; doi:10.1007/s00401-019-02010-2)

## Slide 1
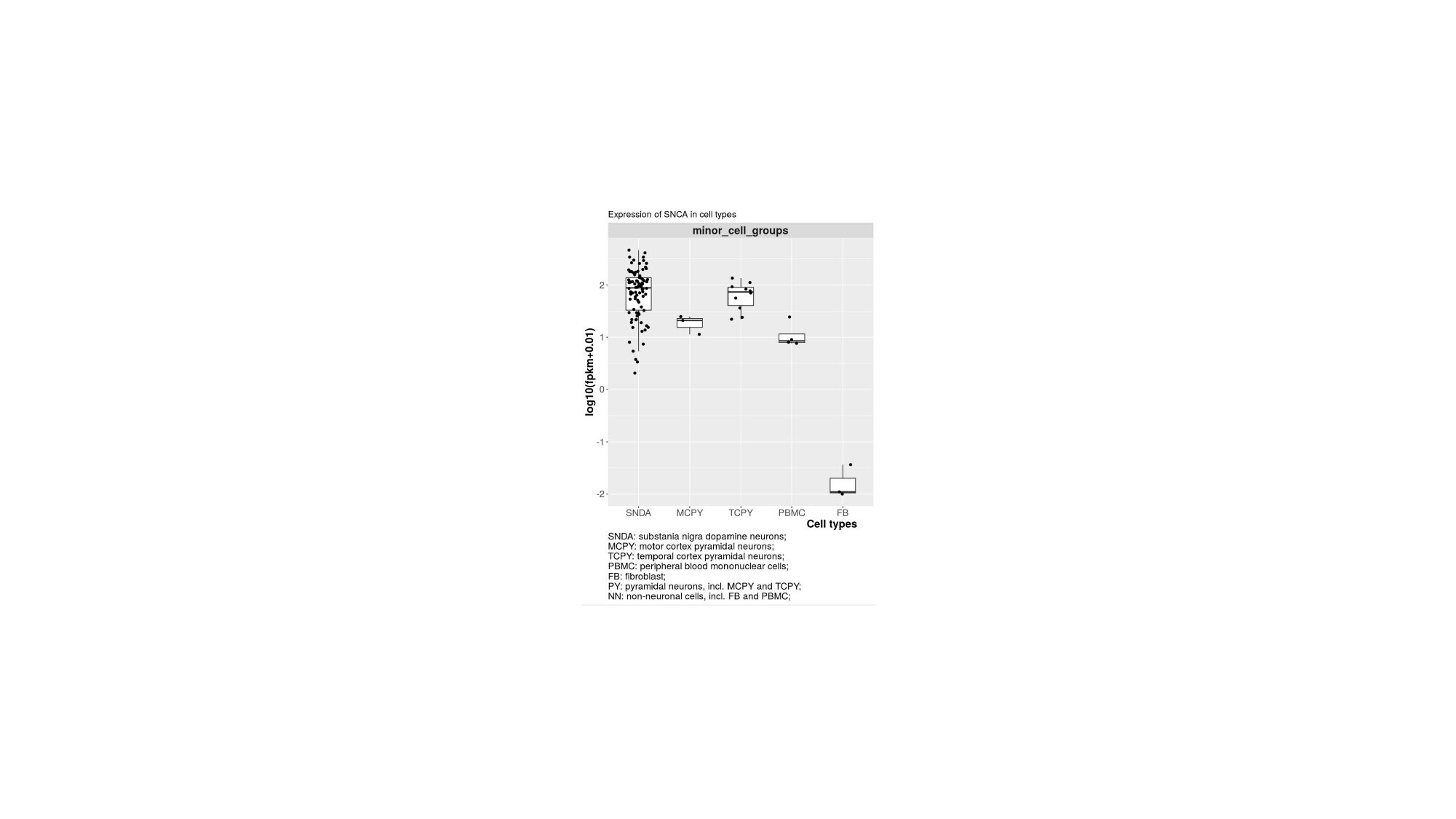

Supplement: Supplementary file 1 — Suppl. Figure 1. BRAINcode Project expression data of SNCA in human laser-captured cells. This analysis shows the expression of SNCA in the substantia nigra DAergic neurons and other cells. SNDA: substantia nigra DAergic neurons, MCPY: motor cortex pyramidal neurons, TCPY: temporal cortex pyramidal neurons, PBMC: peripheral blood mononuclear cells, FB: fibroblasts. (PPTX 160 kb) [file 401_2019_2010_MOESM1_ESM.pptx]
